# Supplementary material for: Splice-Junction-Based Mapping of Alternative Isoforms in the Human Proteome
Source: Cell Rep. Author manuscript; Available in PMC 2020 Jan 15. (PMC6961840; doi:10.1016/j.celrep.2019.11.026)
Supplement: 2 [file NIHMS1546469-supplement-2.zip › DF1/PXD006675/VentricularSeptum/VentricularSeptum_9_TNS1_ALGLKPHNPADILLHPTGEEDEGK.pdf]

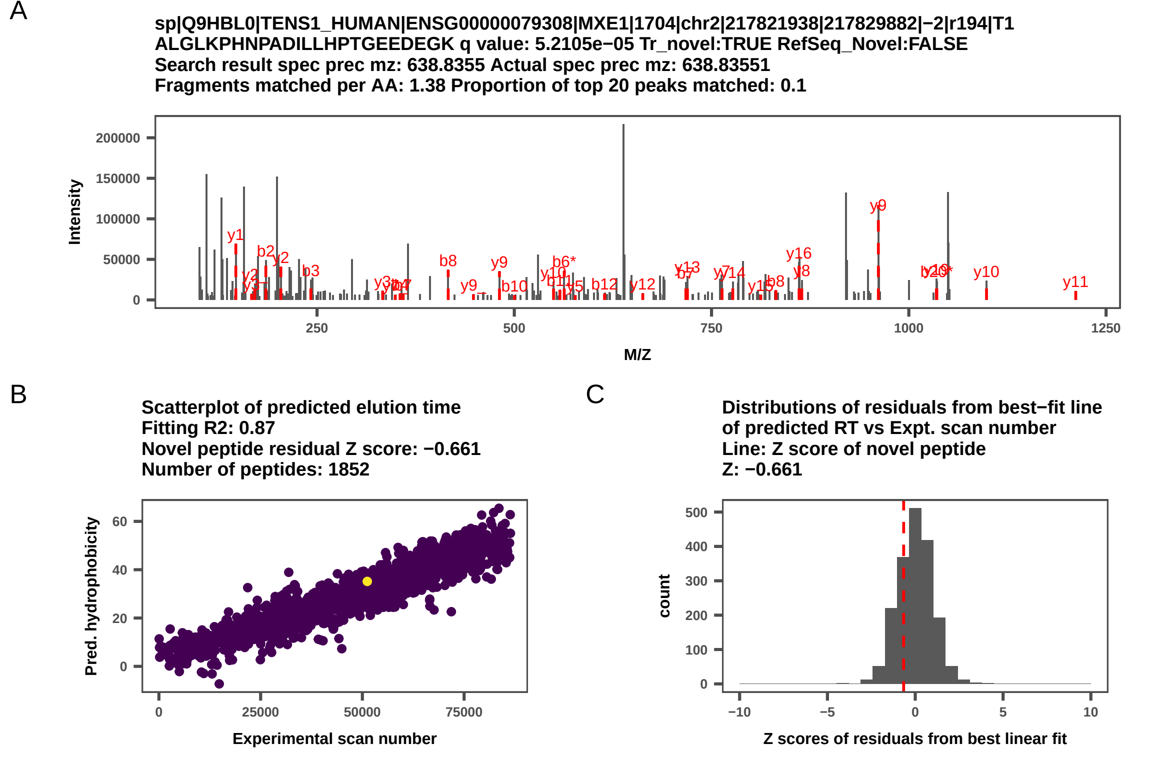
A
sp|Q9HBL0|TENS1_HUMAN|ENSG00000079308|MXE1|1704|chr2|217821938|217829882|−2|r194|T1
ALGLKPHNPADILLHPTGEEDEGK q value: 5.2105e−05 Tr_novel:TRUE RefSeq_Novel:FALSE
Search result spec prec mz: 638.8355 Actual spec prec mz: 638.83551
Fragments matched per AA: 1.38 Proportion of top 20 peaks matched: 0.1
Intensity
M/Z
B
Scatterplot of predicted elution time
Fitting R2: 0.87
Novel peptide residual Z score: −0.661
Number of peptides: 1852
Pred. hydrophobicity
Experimental scan number
C
Distributions of residuals from best−fit line
of predicted RT vs Expt. scan number
Line: Z score of novel peptide
Z: −0.661
count
Z scores of residuals from best linear fit
